# The Concern of COVID-19 Vaccine Safety Is behind Its Low Uptake among Patients with Diabetes Mellitus in Sudan

**DOI:** 10.3390/vaccines10040527

**Published:** 2022-03-29

**Authors:** Saeed M. Omar, Rehana Khalil, Ishag Adam, Osama Al-Wutayd

**Affiliations:** 1 Department of Medicine, Faculty of Medicine, Gadarif University, Gadarif 32211, Sudan; drsaeedomar@gaduniv.edu.sd; 2 Department of Family and Community Medicine, Unaizah College of Medicine and Medical Sciences, Qassim University, Unaizah 56219, Saudi Arabia; rn.noman@qu.edu.sa; 3 Department of Obstetrics and Gynecology, Unaizah College of Medicine and Medical Sciences, Qassim University, Unaizah 56219, Saudi Arabia; ia.ahmed@qu.edu.sa

**Keywords:** COVID-19 vaccine, vaccine uptake, diabetes mellitus, Sudan

## Abstract

Diabetic patients are vulnerable to developing severe complications and have a higher risk of death due to COVID-19 infection. Vaccination remains the mainstay during the current situation to mitigate the risks related to COVID-19 infection. Therefore, the aim of the current study was to assess the vaccination status and the factors associated with COVID-19 vaccine uptake among patients with diabetes mellitus (DM) in Sudan. A hospital-based cross-sectional study was conducted from January to February 2022 at Gadarif Hospital in eastern Sudan. Information on sociodemographics, the contracting of COVID-19 during the pandemic, beliefs toward COVID-19 vaccinations, and barriers related to COVID-19 vaccinations was obtained through an interview questionnaire among adult (≥18 years) patients with DM. Bivariate and multinomial logistic regression analyses performed. A total of 568 diabetic patients were enrolled, with a mean (SD) age of 53.07 (12.69) years. The majority of the participants were female (67.6%), urban residents (63.4%), uneducated (60.6%) and employed (73.2%). There was a history of COVID-19 in 97.4% of participants, and 29.2% of them had hypertension along with DM. About 31% received the vaccine, out of which 17.9% received the first dose, 13.2% received the second dose, and 0.2% received the third dose. Multinomial logistic regression analysis showed a significant association between belief in the safety of the COVID-19 vaccine and having had two doses of it (adjusted Odds ratio = 20.42, *p* < 0.001). The prevalence of COVID-19 infection was high, while the rate of COVID-19 vaccination uptake was low and inadequate among the participants. Appropriate health education and targeted interventions toward awareness of safety concerns are highly recommended.

## 1. Introduction

Since the beginning of the current pandemic, around 395 million confirmed cases of COVID-19 and more than five million deaths have been reported worldwide [1]. The most valuable intervention to limit its spread and/admissions is protective measures along with vaccination [2]. Four types of vaccines with varying safety and efficacy are presently available, including RNA/DNA vaccines, viral vector vaccines, protein-based vaccines, and inactivated virus vaccines [3,4,5,6,7,8,9,10,11]. However, a high vaccine acceptance and uptake rate is required for the success of COVID-19 immunization among the population [12]. Since people living with pre-existing chronic diseases are vulnerable to developing severe complications and are thus at greater risk of death due to COVID-19 infection, the COVID-19 vaccine is highly recommended for them [13,14,15]. Diabetes mellitus (DM) is a chronic disease associated with severe illness, intensive care unit admissions, and high mortality in patients with COVID-19 infection [16,17,18,19,20,21,22]. Moreover, DM and hypertension often occur together [23]. Certain populations are at higher risk of developing type 2 DM, as it is three times more common among Africans and those of African-Caribbean origin [24]. It is estimated that about 14 million people in Africa are affected by DM, and this burden is expected to rise to 28 million by the year 2030 [25]. Evidence shows that seven countries in the World Health Organization (WHO) Eastern Mediterranean region have a prevalence of more than 15%, while seven countries, including Sudan, have a prevalence of 9–12% of diabetes mellitus [26]. It is emerging as one of the serious health problems in the urban population of Sudan and other African countries, with a subsequent increase in hospital admissions and mortality [27]. The major predisposing factor for diabetes is obesity, which has significantly increased in the last two decades due to growing urbanization and altered lifestyles like increased use of vehicles, little exercise, sedentary hours watching TV and the huge consumption of fat, sugar, and refined cereals. Ironically, high healthcare costs contributes to the delayed diagnosis of diabetes, hospital care, and the appearance of complications [28]. Sudan’s healthcare system is very ill-equipped to respond towards the growing and neglected needs of the population of Sudan. There is an inequity in the distribution of health facilities with several underserved areas and a lack of access to basic health services [29].

Sudan is one of the largest African countries, with an area of 1.886 million km^2^ and a population of 40.53 million multi-ethnic Africans and Afro-Arabs [30]. The country has a lower middle economy and its health expenditures are approximately 6.5% of its gross domestic product (GDP), and 8.2% of general government [31] expenditures. Almost 41% of its population is under the age of 15, and 20% are between 15 to 24 years old. Around 47% of the country’s population earn less than $1.25 per day. About 63% of Sudan’s land area is agricultural land, and most of the families depend on small-scale agriculture for their survival [32].

There has been a steady rise in COVID-19 cases since 12 March 2020, when the first case was identified in the country [33]. Until now, Sudan has declared a total of 59,294 confirmed cases of COVID-19, with 3632 deaths. A total of 4,991,228 COVID-19 vaccine doses have been administered in Sudan as of Feb 2022 [34]. A preliminary analysis by the World Health Organization (WHO) has shown that death rates from COVID-19 infections are significantly higher in patients with diabetes in Africa [35]. Contemporary studies have reported that people with type 1 diabetes (T1D) and type 2 diabetes (T2D) have an increased risk of developing severe illnesses from COVID-19 compared with people without DM [36,37]. Both patients with T1D and T2D had analogous adjusted odds ratios (ORs) for the severity of illness (3.35 vs. 3.42), hospitalization (3.90 vs. 3.36), and mortality (3.51 vs. 2.02) [38,39]. Also, a study from eastern Sudan reported a high mortality rate among diabetic patients with COVID-19 [40].

Primary prevention through vaccination remains the mainstay for mitigating the risks related to COVID-19 infection in patients with DM, and thus it is essential to determine the rate of COVID-19 vaccine uptake among diabetic patients and to understand the factors associated with it. Moreover, there is a paucity of published data on DM and COVID-19 vaccinations in Sudan. Therefore, this is the first study aimed at assessing vaccination status and the factors associated with COVID-19 vaccine uptake among people living with DM in Sudan.

## 2. Methods

### 2.1. Study Design, Setting, and Subjects

**Figure 1:**
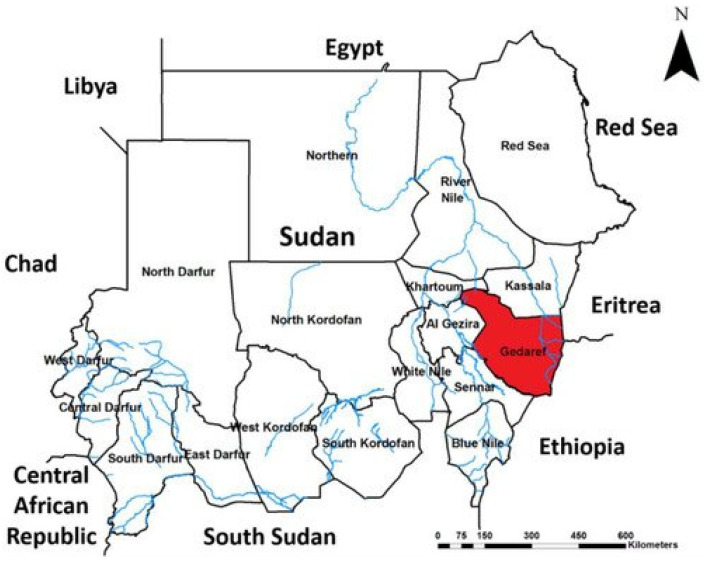
Ref. [43] shows the map of Sudan with highlighted study setting (Gadarif).

A cross-sectional study was conducted on adults with DM at Gadarif Hospital, Sudan, from January to February 2022. Gadarif diabetic Center is situated in the city of Gadarif. It provides outpatient services to all registered or referred diabetic patients of Gedarif state [41]. Gaedaref, also known as El-Gedaref or Al-Qaḍārif, is one of 18 states of Sudan, with an area of 75,263 km^2^, and is located in southeastern Sudan roughly between the latitudes 14 and 16 North and longitudes 35 and 36 East in the semi-desert tropics [42]. Its total population is 1,827,181 and includes mainly Arab or Nubian Sudanese, including indigenous Beja [43]. Most of them are nomads, refugees from neighbour countries of Ethiopia, Eritrea, and West African countries, and internally displaced persons (IDPs). Farming, trading, and animal breeding are the basic sources of their income [44] Figure 1 [43].

The vaccine was freely available for all diabetes patients in sufficient quantity at the study site with no barrier to limit its availability.

### 2.2. Sample Size Calculation

The sample size was calculated through OpenEpi software and the minimum sample size required for this study was 380 diabetics considered with 5% precision and 95% confidence, and the rate of vaccine uptake among diabetics was 55.5% [45].

## 3. Data Collection

Data were collected by a trained nurse via face-to-face interviews. The investigators developed a questionnaire based on recently published information to address the study’s objectives [46,47]. A pilot study on 20 subjects was conducted and was not included in the results. The questionnaire was reviewed by three experts to ensure face and content validity.

### 3.1. Variables of Interest

#### 3.1.1. Outcome Variable

In our study, the dependent variable was categorized into four classes according to the number of COVID-19 doses (not vaccinated, vaccinated with the 1st dose, full vaccination, vaccinated with booster dose).

#### 3.1.2. Explanatory Variables

Sociodemographic data: age, gender (male/female), place of residence (urban/rural), level of education (uneducated, secondary school, university and above), occupation (unemployed/employed), diagnosed with hypertension (yes/no).

Information about contracting COVID-19 during pandemic: history of COVID-19 infection (yes/no), and did any one of your contacts suffer from COVID-19? (yes/no).

Beliefs toward COVID-19 vaccination: Do you think that the COVID-19 vaccine is safe? (yes, no, not sure), Do you think that the COVID-19 vaccine is effective? (yes/no/not sure).

Do you think that the best way to avoid the complications (hospitalization and death) of COVID-19 is by getting the vaccine? (yes, no, not sure).

Barriers related to COVID-19 vaccination: concern about side effects, belief that the vaccine will not prevent infection, conspiracy theory, etc. Also include conditions that may encourage them to get the COVID-19 vaccine, such as if my physician recommended it, if it is mandatory for continuing in my job, compulsory by the government, if my family or friends get vaccinated, I will not take it under any condition, or other (select only one answer).

### 3.2. Statistical Analysis

The data were entered into an Excel spreadsheet and exported to STATA version 16.0 for statistical analysis. The data were presented as a number and percentage for categorical variables or as mean and standard deviation (SD) for continuous variables according to the dependent variable. Continuous variables were assessed using the unpaired *t*-test, while a chi-squared test was used to assess the categorical variables. Multinomial logistic regression analysis was performed to find the association of independent variables with dependent variables (vaccinated with the 1st dose, full vaccination “two doses”, not vaccinated as a reference category, and vaccinated with booster dose were not included in the analysis due to insufficient data). Adjusted odds ratios (aORs) with 95% confidence intervals (CIs) are reported. A *p* value of <0.05 was considered to be strong evidence against the null hypothesis.

## 4. Results

A total of 568 diabetic patients participated in the study. The mean age of the participants was 53.07 (12.69) years, and the majority (*n* = 384, 67.6%) of them were female. Most (*n* = 360, 63.4%) of the participants were urban residents, (*n* = 344, 60.6%) were uneducated and were (*n* = 416, 73.2%) employed. A large proportion (*n* = 553, 97.4%) of the study participants had a history of COVID-19, and less than one-third (*n* = 166, 29.2%) had hypertension along with DM (Table 1). The mean age of the patients who received no dose (34.2 ± 12.5), one dose (33.2 ± 11.9) or two doses (33.8 ± 12.9) was not statistically different. Further details of sociodemographics according to the dependent variable are presented in Table 1. The bivariate analysis shown in Table 2 revealed that the statistical difference is significant regarding the concern of vaccine safety (*p* < 0.001), effectiveness (*p* < 0.001), the best way to avoid the complications of COVID-19 (*p* < 0.001) and the employment status (0.024) of the participants. Table 3 presents a multinomial logistic regression analysis, showing that the diabetic patients who believed that it was safe for them were more likely (aOR 20.42, 95% CI *p* < 0.001) to have two doses, i.e., a “full vaccination” of the COVID-19 vaccine, as compared to those who did not take the vaccine. The most common barrier to vaccine uptake among diabetics included concerns about vaccine side effects (*n* = 235, 60%). Some (*n* = 64, 16.37%) participants believed that vaccines cannot save them from infection, while others (*n* = 38, 9.72%) were of the opinion that there were conspiracy theories about vaccines. Other reasons included fear of needles/syringes (*n* = 15, 3.84%), the perceived safety of the participants from infection due to their young age and good health status (*n* = 13, 3.32%), and fulfillment of precautionary measures (*n* = 26, 6.65%). Almost half (*n* = 210, 53.7%) of the 568 participants were ready to receive the vaccine if it was recommended by their physicians. Other responses included that they would get the vaccine if the vaccine was mandatory for continuation of their job (*n* = 21, 5.37%), if it was compulsory by the government (MOH) (*n* = 22, 5.63%), if their family or friends get vaccinated (*n* = 32, 8.18%), if research studies showed that the vaccine is safe and effective (*n* = 37, 9.46%), or if there was a mode other than injection, such as an oral vaccine (*n* = 4, 1.02%). However, there was also a proportion (*n* = 65, 16.62%) of participants who were not ready to receive the vaccine under any condition.

## 5. Discussion

Our results indicate that vaccine uptake among diabetic patients in Sudan is low. About one-third of the respondents reported an uptake of the COVID-19 vaccine, and only 0.2% of them received a third dose (booster dose). This finding is quite undesirable because diabetic patients are considered to be a high-risk population due to the established high prevalence of complications and adverse outcomes of COVID-19 infection compared to nondiabetic subjects [48,49,50,51]. A study has reported an 81% mortality rate among diabetes patients with severe COVID-19 infection as compared to 48% in people without diabetes [52]. Moreover, the intensive care unit admission risk for diabetic patients with COVID-19 infection is 14.2%, more than for people without diabetes [53]. Additionally, a booster dose is highly recommended for high-risk groups, including those with chronic diseases such as diabetes mellitus [54]. However, the total rate of vaccine uptake in our study is higher than the rate (21.5%) reported by a contemporary study in India, in which 17% of participants received one dose, and 4.2% received two doses [55]. The proportion of vaccine uptake among diabetics in this study is almost similar to a recent study conducted in Saudi Arabia, which demonstrated uptake of 34.7% among diabetes patients [56]. Nevertheless, an Italian survey showed that 5.1% of patients had received a COVID-19 vaccination at the time of the survey, but overall, 77.9% were willing to get vaccinated [57]. It is noteworthy that about two-thirds (69%) of our study participants did not receive any dose of the vaccine, which is the highest among all published studies on COVID-19 vaccine hesitancy among diabetic patients, including 29% in Saudi Arabia, 14.2% to 18.3% in Italy, and 56.4% in China [56,57,58,59]. This necessitates more focused efforts to motivate the people living with diabetes in Sudan and to make them aware of the safety and efficacy of the COVID-19 vaccine because the safety, effectiveness and ability of the available vaccine to protect against complications of this infection are some factors that are identified to increase the likelihood of vaccine acceptance among the study participants. There was also some evidence of an association between those who received two doses (*p* = 0.096) and the employment status of the participants as compared to those who were unemployed. However, no evidence of an association between gender and educational status with vaccine uptake was found in this study, unlike the previous studies conducted among different countries and populations, which showed an association of higher education and males with vaccination uptake [60,61,62,63]. In Sudan, the AstraZeneca vaccine was deployed for the vaccination of the population after receiving a large number of vaccine doses in March 2021 through COVAX and UNICEF [64]. This study revealed that the main barrier to getting vaccinated among study respondents was their concerns about the side effects of the vaccine, and this response accounted for more than half of participants who did not receive the vaccine. Predictably, one of the reasons for hesitancy to receive COVID-19 vaccines among a high-risk population was driven by doubts about an expedited process of approval along with immediate and long-term safety concerns, as well as recently published reports on coagulopathy associated with COVID-19 vaccinations, particularly the ChAdOx1 nCoV-19 (Oxford–AstraZeneca) vaccine [46,65,66,67,68]. Over and above that, only short-term effects of COVID-19 vaccine are noted, without consideration of long-term effects, which are still unknown, and there is still a suspicion that vaccine-related complications exceed the COVID-19 infection risk itself [69]. Other barriers identified in the current study included a belief that a vaccine would not save our participants from infection, conspiracy theories about the vaccine, fear of needles/syringes, perceived protection from infection due to their young age, good health, and fulfillment of precautionary measures. Similar results were shown by Alghamdi et al. in their study in Saudi Arabia about acceptance and barriers of the COVID-19 vaccine among people with chronic diseases, where about 55% of hesitant respondents expressed their concerns about the side effects and almost 48% were not vaccinated because of their belief that preventive precautions could suffice, and participants also communicated their fear of needles as one of the reasons [69]. Likewise, reluctance due to side effects of the vaccine was observed to be the prime barrier in a Chinese study by Wang et al. [59]. Our findings of conspiracy theories related to the COVID-19 vaccine are supported by several studies [56,70,71]. A global-level cross-sectional study demonstrated a significant relationship between misinformation about vaccines and the rate of vaccination uptake [72]. With regard to our finding about belief of the respondents that the vaccine cannot save them from SARS-CoV-2, there is a limited availability of evidence addressing the immune response among diabetic patients with COVID-19, which provides a rationale for the effectivity of vaccination against SARS-CoV-2, including a study by Dispinseri et al. in Italy which found that diabetes (hyperglycemia) has no effect on kinetics and neutralizing antibody response is needed against the SARS-CoV-2 spike protein, which results in a reduction in the mortality among patients with and without diabetes mellitus [73]. Another Italian study by Lampasona et al. demonstrated the humoral immune response among diabetic patients against SARS-CoV-2 [74].

Furthermore, with regard to the investigated conditions on which the hesitant participants of our study were willing to get a vaccine, the results showed that more than half replied that they would get it if their doctors would recommend it for them. This finding is in agreement with the aforementioned study, which showed that the majority of unwilling diabetics were ready to receive vaccines on their diabetologist’s recommendation [55]. Since the majority of our participants had safety concerns and they trusted their doctors’ advice, it is highly recommended to maximize the vaccination campaign for diabetic patients through a high-risk approach involving physicians and diabetologists in an organized and targeted intervention toward awareness. The strength of the current study is that it is the first study with a sufficiently large sample size to investigate the rate of COVID-19 vaccine coverage among diabetics. Our results provide practical implications for policymakers to increase the rate of vaccine uptake among diabetic patients by launching targeted public awareness campaigns for those diabetic patients with chronic misconceptions regarding the safety and efficacy of the vaccine. Although the study presented valuable information, it had some limitations. First, it was a cross-sectional study, so the causal relationship between factors and outcome variables could not be analyzed. Second, the study was conducted in one hospital and thus could not be said to be representative of the country. Third, there is limited accessibility of Sudan’s population to healthcare services due to low governmental expenditures on the health sector. It is estimated that the out-of-pocket share of the Sudanese population is about 70% (US$84.0 per capita) and government health expenditure consists of only 22.3% (US$26.9 per capita) [31]. Also, factors including the country’s political difficulties, harsh weather, decades of mismanagement and conflicts with displaced people and refugees have a considerable impact on its healthcare sector [75]. Therefore, the study may have underestimated the gap of vaccine availability to lower income patients.

## 6. Conclusions

The prevalence of COVID-19 infection was high among participants, while the rate of COVID-19 vaccination uptake was low and inadequate. Appropriate health education and targeted interventions toward awareness of safety concerns are highly recommended.

## Figures and Tables

**Table 1:** Sociodemographic characteristics of the participants in Sudan according to the number of COVID-19 vaccine doses (*n* = 568).

Characteristics	Total	Not Vaccinated *n* (%)	Vaccinated with the 1st Dose *n* (%)	Full Vaccination *n* (%)	Vaccinated with Booster Dose *n* (%)
		390 (68.7)	102 (17.9)	75 (13.2)	1 (0.2)
Age, mean [SD]	53.07 [12.69]	34.2 [12.5]	33.2 [11.9]	33.8 [12.9]	24 (0)
Sex					
Female	384 (67.6)	273 (71)	67 (14.5)	43 (11.2)	1 (03)
Male	184 (32.4)	117 (63.6)	35 (19)	32 (17.4)	0
Residence					
Urban	360 (63.4)	245 (68)	67 (18.6)	48 (13.3)	0
Rural	208 (36.6)	145 (69.7)	35 (16.8)	27 (13)	1 (0.5)
Education level					
Uneducated	344 (60.6)	246 (71.5)	60 (17.4)	37 (10.8)	1 (0.3)
Secondary	174 (30.6)	110 (63.2)	33 (19)	31 (17.8)	0
University and above	50 (8.8)	34 (68)	9 (18)	7 (14)	0
Occupation					
Unemployed	152 (26.8)	93 (61.2)	30 (19.7)	29 (19.1)	0
Employed	416 (73.2)	297 (71.4)	72 (17.3)	46 (11.1)	1 (0.2)
History of COVID-19 infection					
No	15 (2.6)	380 (68.7)	100 (18.1)	72 (13)	1 (0.2)
Yes	553 (97.4)	10 (66.7)	2 (13.3)	3 (20)	0
Hypertension					
No	402 (70.8)	282 (70.2)	72 (17.9)	48 (11.9)	0
Yes	166 (29.2)	108 (65.5)	30 (18.2)	27 (16.4)	1 (0.6)

**Table 2:** Bivariate analysis of factors associated with frequency of COVID-19 vaccine doses among diabetic patients (*n* = 567).

Variables	Not Vaccinated	Vaccinated with the 1st Dose	Full Vaccination	*p* Value
	Mean (SD) was compared using unpaired *t* test			
Age, years	34.2 (12.5)	33.2 (11.9)	33.8 (12.9)	0.787
	Frequency (%) were compared using chi-square test			
Sex				
Female	273 (71)	67 (14.5)	43 (11.2)	0.091
Male	117 (63.6)	35 (19)	32 (17.4)	
Residence				
Urban	245 (68)	67 (18.6)	48 (13.3)	0.862
Rural	145 (70.1)	35 (16.9)	27 (13)	
Education level				
Uneducated	246 (71.5)	60 (17.4)	37 (10.8)	0.223
Secondary	110 (63.2)	33 (19)	31 (17.8)	
University and above	34 (68)	9 (18)	7 (14)	
Occupation				
Unemployed	297 (71.6)	72 (17.4)	46 (11.1)	0.024
Employed	93 (61.2)	30 (19.7)	29 (19.1)	
Hypertension				
No	282 (70.2)	72 (17.9)	48 (11.9)	0.348
Yes	108 (65.5)	30 (18.2)	27 (16.4)	
History of COVID-19 infection				
No	380 (68.7)	100 (18.1)	72 (13)	0.694
Yes	10 (66.7)	2 (13.3)	3 (20)	
Did anyone in your contacts suffer from COVID-19 during this pandemic?				
No	357 (68.5)	93 (17.9)	71 (13.6)	0.634
Yes	33 (71.7)	9 (19.6)	4 (8.7)	
Do you think that the COVID-19 vaccine is safe?				
No/not sure	182 (94.3)	9 (4.7)	2 (1)	<0.001
Yes	208 (55.6)	93 (24.9)	73 (19.5)	
Do you think that the COVID-19 vaccine is effective?				
No/not sure	197 (91.6)	11 (5.1)	7 (3.3)	<0.001
Yes	193 (54.8)	91 (25.9)	68 (19.3)	
Do you think that the best way to avoid the complications of COVID-19 is by getting the vaccine?				
No/not sure	194 (91.1)	12 (5.6)	7 (3.3)	<0.001
Yes	196 (55.4)	90 (25.4)	68 (19.2)	

**Table 3:** Multinomial logistic regression analysis of independent variables associated with getting one and two doses of COVID-19 vaccine.

Variables	Vaccinated with the 1st Dose		Full Vaccination	
	OR (95% CI)	*p*-Value	OR (95% CI)	*p*-Value
Sex				
female	Reference		Reference	
Male	1.19 (0.71, 1.99)	0.517	1.49 (0.84, 2.66)	0.176
Education level				
Uneducated	Reference		Reference	
Secondary school	0.95 (0.57, 1.60)	0.856	1.29 (0.72, 2.29)	0.383
University and above	0.76 (0.33, 1.76)	0.521	0.87 (0.33, 2.24)	0.767
Occupation				
Unemployed	Reference		Reference	
Employed	1.21 (0.69, 2.09)	0.505	1.67 (0.91, 3.05)	0.096
Do you think that the COVID-19 vaccine is safe?				
No	Reference		Reference	
Yes	2.84 (0.87, 9.19)	0.082	20.42 (3.79, 109.73)	<0.001
Do you think that the COVID-19 vaccine is effective?				
No	Reference		Reference	
Yes	2.02 (0.55, 7.39)	0.288	0.76 (0.21, 2.77)	0.677
Do you think that the best way to avoid the complications of COVID-19 is by getting the vaccine?				
No	Reference		Reference	
Yes	2.07 (0.73, 5.83)	0.171	2.37 (0.71, 7.89)	0.161

Note: not vaccinated as a reference category.

## Data Availability

The data presented in this study are available on request from the corresponding author.
